# Role of Atmospheric Indices in Describing Inshore Directional Wave Climate in the United Kingdom and Ireland

**DOI:** 10.1029/2020EF001625

**Published:** 2021-05-05

**Authors:** T. Scott, R. J. McCarroll, G. Masselink, B. Castelle, G. Dodet, A. Saulter, A. A. Scaife, N. Dunstone

**Affiliations:** ^1^ School of Biological and Marine Sciences University of Plymouth Plymouth UK; ^2^ UMR EPOC University of Bordeaux/CNRS Bordeaux France; ^3^ IFREMER CNRS IRD Laboratoire d'Océanographie Physique et Spatiale IUEM University of Brest Brest France; ^4^ UK Met Office Exeter UK; ^5^ College of Engineering, Mathematics and Physical Sciences University of Exeter Exeter UK

**Keywords:** climate indices, coastal evolution, inshore wave climate, long term prediction, seasonal forecasting, wave direction

## Abstract

Improved understanding of how our coasts will evolve over a range of time scales (years‐decades) is critical for effective and sustainable management of coastal infrastructure. A robust knowledge of the spatial, directional and temporal variability of the inshore wave climate is required to predict future coastal evolution and hence vulnerability. However, the variability of the inshore directional wave climate has received little attention, and an improved understanding could drive development of skillful seasonal or decadal forecasts of coastal response. We examine inshore wave climate at 63 locations throughout the United Kingdom and Ireland (1980–2017) and show that 73% are directionally bimodal. We find that winter‐averaged expressions of six leading atmospheric indices are strongly correlated (*r* = 0.60–0.87) with both total and directional winter wave power (peak spectral wave direction) at all studied sites. Regional inshore wave climate classification through hierarchical cluster analysis and stepwise multi‐linear regression of directional wave correlations with atmospheric indices defined four spatially coherent regions. We show that combinations of indices have significant skill in predicting directional wave climates (*R* ^*2*^ = 0.45–0.8; *p* < 0.05). We demonstrate for the first time the significant explanatory power of leading winter‐averaged atmospheric indices for directional wave climates, and show that leading seasonal forecasts of the NAO skillfully predict wave climate in some regions.

## Key Points

- Over 70% of inshore wave climates analyzed throughout the United Kingdom and Ireland were directionally bimodal
- Combinations of winter atmospheric indices NAO, WEPA, SCAND, and EA are significantly correlated with directional wave climate in all regions
- Regression models using multiple winter atmospheric indices enable skillful reconstructions of directional wave climate in all regions

## 1 Introduction

Improved understanding and awareness of how our coasts and beaches will evolve over a range of time scales (years to decades) is critical for effective and sustainable management of our coastal infrastructure. While coastal erosion is already a problem globally (Luijendijk et al., 2018; Mentaschi et al., 2018), climate change can also affect the primary drivers of coastal change, impacting local sea‐level changes (Cazenave et al., 2014) and increased storminess in some regions of the world (Scaife, Spangehl, et al., 2012; Zappa et al., 2013). Globally, increased sea‐level rise will likely result in increased erosion (Le Cozannet et al., 2016), and increased frequency and intensity of coastal flooding along low‐lying coasts (e.g., Vousdoukas et al., 2018). Regionally, atmospheric processes described by teleconnection patterns like the North Atlantic Oscillation (NAO) in Europe, can modulate sea‐level and wave climate, impacting coastal change (Tsimplis et al., 2005). Advances in understanding of current and future shoreline change and coastal vulnerability require robust knowledge of the forcing wave climate, including spatial, directional, and temporal variability at any given location. The advent of long‐term (multi‐decadal) atmospheric sea‐level pressure records (e.g., Antolínez et al., 2018; Poli et al., 2016), hindcast directional wave timeseries (e.g., Dodet, Bertin, & Taborda, 2010) and beach morphological records (e.g., Ludka et al., 2019; Masselink et al., 2016; Turner et al., 2016) have provided fresh insights into the relationships between multi‐decadal atmospheric variability, inshore wave climate and beach morphological response.

An examination of long‐term beach morphological data sets throughout exposed western coasts of Europe by Dodet, Castelle, et al. (2018) highlights the important role of winter‐averaged wave conditions in controlling local shoreline response in regions that are dominated by cross‐shore exchange (on‐offshore) of beach sediments on seasonal and greater timescales. Woolf, Challenor, and Cotton (2002) and Woolf, David Cotton, and Challenor (2003) and more recently Castelle, Dodet, Masselink, and Scott (2017) have demonstrated that winter‐averaged atmospheric indices, specifically the North Atlantic Oscillation (NAO; Hurrell, 1995), and the newly developed West Europe Pressure Anomaly (WEPA; Castelle, Dodet, Masselink, & Scott, 2017), significantly explain a very large amount of annual winter‐averaged wave height variability on exposed west‐coast sites from Ireland to Portugal. Castelle, Dodet, Masselink, and Scott (2017) showed that the winter‐averaged NAO expressed the strongest relationship north of southern Ireland (52°N) and the winter‐averaged WEPA exhibited the strongest relationship from the south of Ireland to the south of Portugal. Dodet, Bertin, and Taborda (2010) demonstrated for the first time the link between the winter averaged mean wave direction variability and the winter‐averaged NAO in the North East Atlantic, with positive correlations up to 0.7 in South Portugal. Martínez‐Asensio et al. (2016) also examined relationships between the wind wave climate and the main climate modes of atmospheric variability (1989–2007) in the North Atlantic Ocean (including NAO, East Atlantic (EA) pattern, East Atlantic Western Russian (EA/WR) pattern and the Scandinavian (SCAND) pattern), demonstrating that NAO and EA (which has similar characteristics to WEPA) patterns are the most relevant. However, none of these studies have examined these relationships within directionally multimodal wave climates, common to more sheltered and protected seas.

Along the exposed coasts of western Europe facing the dominant wave direction, beach response is largely dominated by on‐offshore cross‐shore sediment transport which is a function of total incident wave power (Burvingt et al., 2018; Castelle, Marieu, et al., 2014; Masselink et al., 2016; Scott et al., 2016). For beaches orientated away from the dominant wave approach, incident wave angles are more oblique and morphological changes are increasingly driven by longshore transport processes, with sediment transported along the shore in the direction of wave approach (Bühler & Jacobson, 2001; Short & Masselink, 1999). Within embayed beach environments, changes in planform orientation, referred to as “rotation” (Klein et al., 2002), and can be driven by either spatial variability of cross‐shore sediment transport, or longshore transport gradients. Harley, Turner, Short, and Ranasinghe (2011) demonstrated that rotation can be linked to subtle variations in alongshore gradients of wave energy, and hence cross‐shore sediment exchange, leading to out‐of‐phase response at embayment extremities. In contrast to this mechanism, along many (semi‐) sheltered environments where wave climate is generally a mix of swell and local wind wave components, the incident wave climate can be directionally bimodal (spanning the shore‐normal). In this case, and for the purposes of this study, directional bimodality is assumed to be asynchronous and relates to the presence of distinct directional modes within the long term mean directional wave climate, rather than synchronous spectral bimodality that describes multiple peaks within the directional power spectrum. In such environments morphological changes can be controlled by the time‐integrated balance of alongshore wave power from the two directions (Bergillos et al., 2016; Ruiz de Alegria‐Arzaburu & Masselink, 2010; Wiggins, Scott, Masselink, Russell, & McCarroll, 2019a). These (semi‐) sheltered rotation‐dominated coastal sites usually exhibit significant inter‐annual directional variability in shoreline alignment, with seasonal rotational phases often leading to erosion and increased coastal vulnerability at one or other end of the embayment (e.g., Scott et al., 2016).

Recent basin‐wide research into inter‐annual wave climate variability in the Pacific (Barnard et al., 2015; Mortlock & Goodwin, 2016) uncovered subtle links between El Nino Southern Oscillation (ENSO) modes and wave direction, and subsequent variability in cross‐shore beach response. Further to this, modeling work by Splinter et al. (2012) and field observations by Harley, Turner, Kinsela, et al. (2017) have highlighted, not only the relationship between climate indices on wave direction and the subsequent impact on shoreline dynamics along the east coast of Australia, but also the impact of storm wave direction on coastal vulnerability along embayed coasts in general. In northwest Europe, research by Tsimplis et al. (2005) indicated that changes in the NAO are likely to impact wave direction at the coast, and Wiggins, Scott, Masselink, Russell, and McCarroll (2019a) show that winter‐averaged variability in NAO and WEPA has significant skill in explaining wave directional balance in regions where wave climate is strongly bi‐directional, as well as driving beach rotation in these regions (Wiggins, Scott, Masselink, Russell, & Valiente, 2019b).

The NAO represents the principle mode of variability in the North Atlantic climate, and the skillful predictability of winter NAO is critical for long‐range forecasting of the European surface winter climate (Wang et al., 2017). As an intrinsic mode of variability in atmospheric circulation, the dynamics associated to the NAO have in the past been considered unpredictable and largely stochastic in nature (Kim et al., 2012; Smith, Scaife, Eade, & Knight, 2016). But recent forecast systems (Dunstone et al., 2016; Scaife, Arribas, et al., 2014) have shown significant skill provided large ensembles are used (Athanasiadis et al., 2016 report correlation skill of 0.86 with large multimodel ensembles) due to the anomalously weak signal‐to‐noise ratio of climate signals (Scaife & Smith, 2018), achieving correlation coefficients of *r* > 0.6 for winter season (DJF) forecasts initiated on November 1. Dunstone et al. (2016) highlighted potential for further improvements in skill through increased ensemble size and decadal predictability of the NAO with large ensembles was recently reported by Smith, Eade, et al. (2019). Advances have also been achieved through empirical approaches to forecasting the NAO. For example, Wang et al. (2017) used multiple linear regression of key discriminant variables (sea‐ice concentration, stratospheric circulation and sea‐surface temperature) and obtained forecast skill (*r*) of 0.69–0.71. Combined, these advances suggest that skillful prediction of seasonal and decadal coastal vulnerability may be possible (Colman et al., 2011; Dobrynin et al., 2019), where forecasts of climate indices may provide a valuable tool for managing risk to society due to extreme winter‐wave events, wave directional variability and corresponding geomorphological change at the coast.

An improved understanding of how leading atmospheric indices can explain seasonal to multi‐decadal variability in wave power and directionality, and consequentially beach state, lays the foundation for: (1) new insights into climate controls on basin‐scale coastal change; and (2) potential exploitation of skillful season ahead and decadal forecasts of atmospheric indices. The overall aim of this paper is to investigate whether climate variability, synthesized by leading winter‐averaged atmospheric indices (NAO, WEPA, EA, EA/WR, SCAND, and the Artic Oscillation AO), significantly controls the directional balance of alongshore wave power at inshore locations throughout the UK & Ireland (UK&I), characterized by directionally bimodal (semi‐) sheltered seas. The specific objectives are to: characterize the directional wave climate of the UK & Ireland (Section 3); examine relationships between winter wave climate and leading atmospheric indices (Section 4), exploring the regional coherence (Section 5); developing multi‐linear regression models for predicting winter directional wave climate (Section 5); and assessing the current skill of season ahead forecasts to create useful predictions for coastal managers (Section 6).

## 2 Data Sets

### 2.1 Wave Modeling

**Figure 1:**
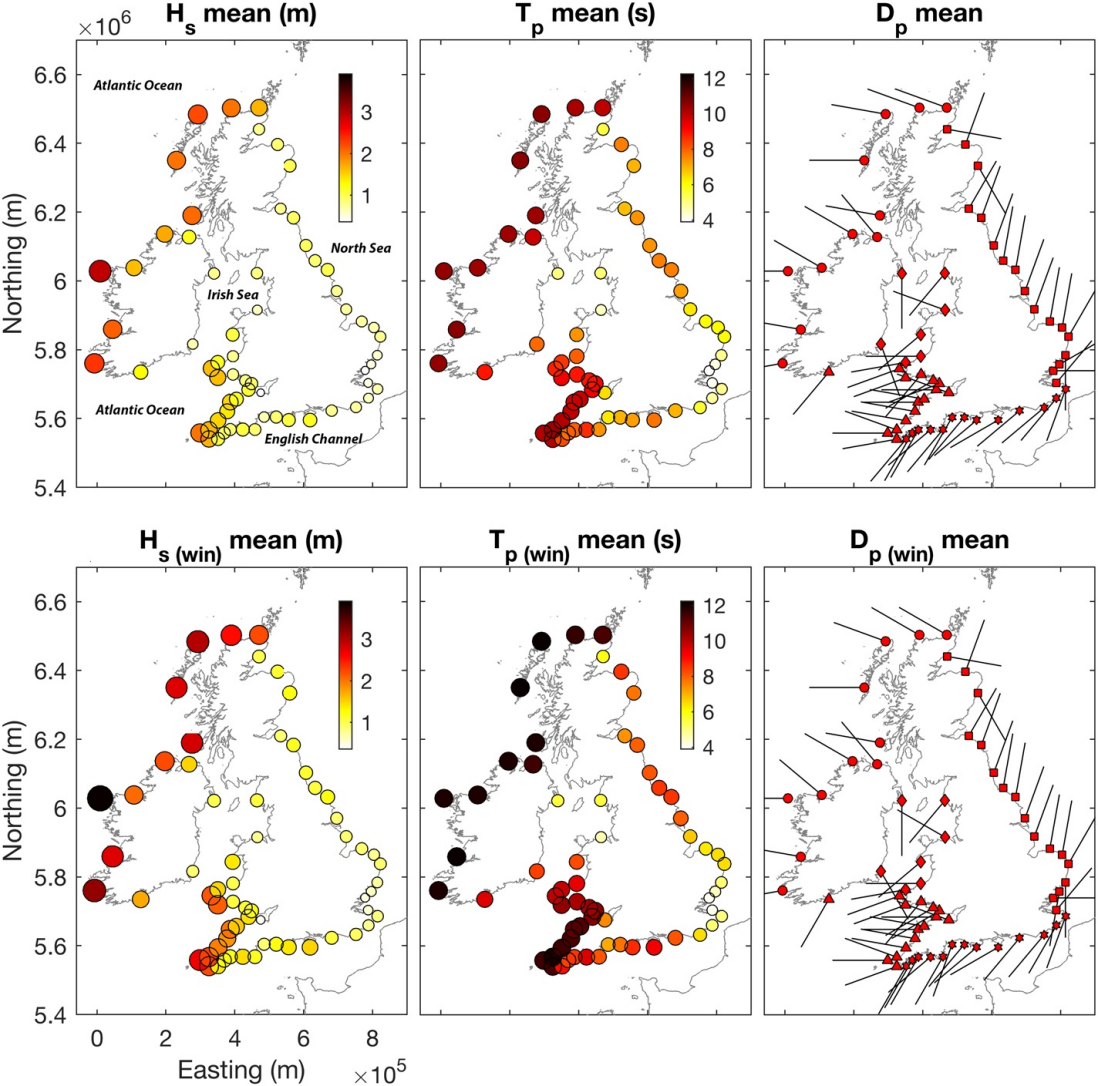
Overview of annual (top panels) and winter (bottom panels) wave climate (1980–2017) around the UK&I coast (all 63 wave model nodes)—mean significant wave height (left panels), mean peak wave period (middle panels), modal peak wave direction (frequency of occurrence; right panels). The size of symbols (left/middle) are proportional to colormap values.

The directional wave climates throughout the UK&I were analyzed at 63 coastal locations (∼20 m depth), hitherto referred to as nodes, using data from the UK Met Office 8‐km WAVEWATCH III third‐generation spectral wave model (version 3.14; Tolman, 2009), representing a 3‐hourly hindcast of integrated wave parameters for the period 1980–2017. The 63 sites were selected to represent all major stretches of exposed coastline throughout the UK&I (Figure 1), and range from the extremely exposed storm‐dominated Atlantic west coasts of Ireland, Scotland and southwest England, to more sheltered locally derived wind‐wave dominated regions in the Irish Sea, North Sea, and English Channel. Section 3 provides an overview of the annual wave climate in each region for 1980–2017.

**Figure 2:**
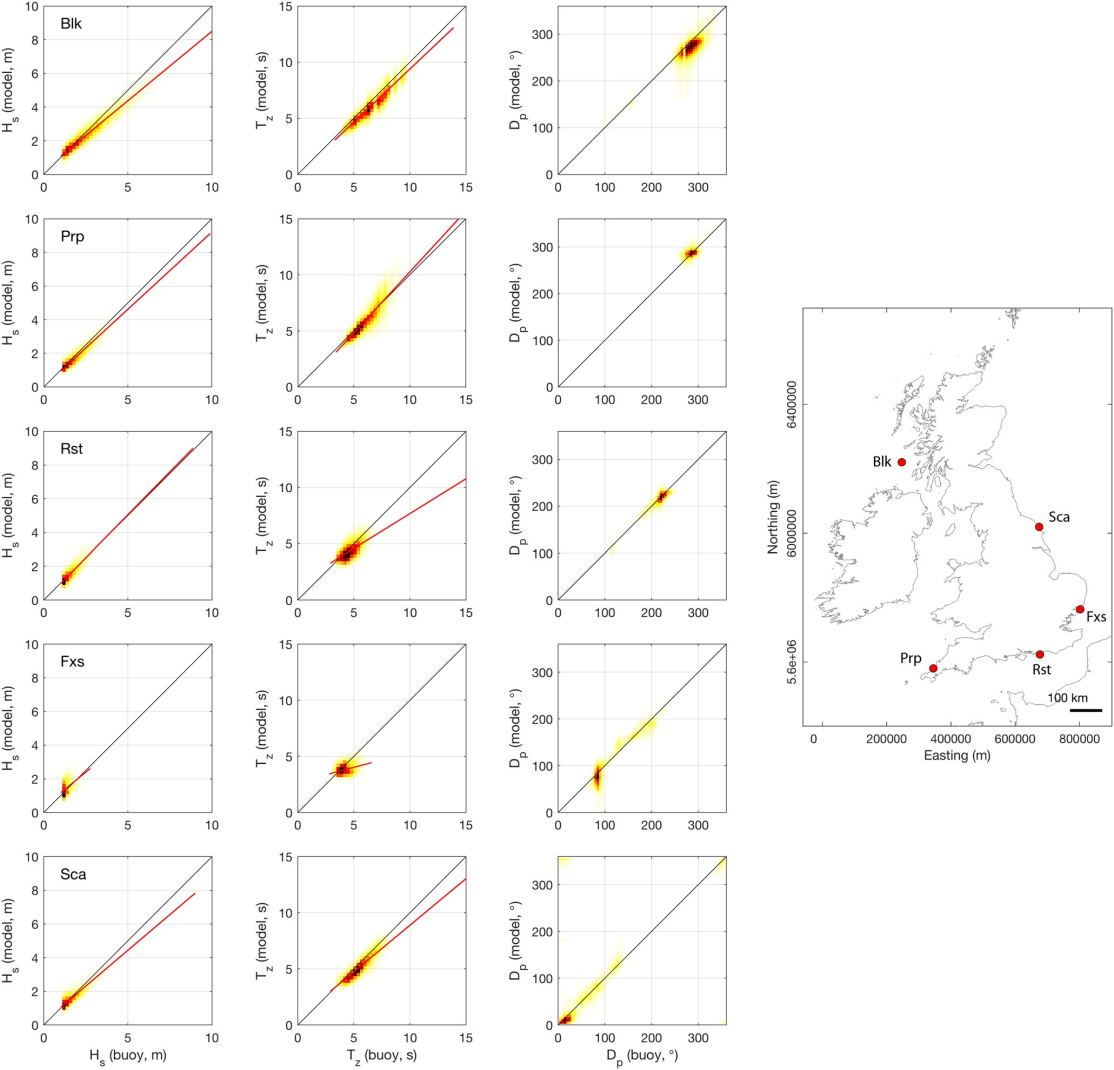
Correlations between nearshore wave buoy observations and modeled wave parameters. Black line is 1:1 fit and red line is linear fit (where appropriate). From left to right columns are: *H*_s_, *T*_z_, and *D*_p_, respectively. From top to bottom rows are locations: Blackstones (Blk), Perranporth (Prp), Rustington (Rst), Felixstowe (Fxs), and Scarborough (Sca). Inset shows locations on map.

The wave model used in the study is described in detail by Mitchell et al. (2017) and has been extensively validated with offshore wave buoys and satellite altimeters by Saulter (2015). A further validation was conducted here to establish model skill at the coastal boundary throughout 5 sites (water depths <20 m with exception of Blackstones (Blk) at ∼90 m) displaying the full range of coastal orientation in the study (exposed to sheltered; Figure 2, Table 1). Modeled waves at the coastal boundary explained 70%–94% of observed variability in significant wave height (*H*_s_), effectively representing bulk energy transfer from the atmosphere. Correlations also show 20%–84% of variability in average zero‐upcrossing wave period (*T*_z_) is explained, representing frequency spread well in all but most sheltered locations (Fxs). A systematic low *T*_z_ bias could be explained by differences in how parameter is represented by model and observations in mixed swell/wind‐sea environments (Saulter, 2018). Importantly, modeled peak wave direction (*D*_p_) explained 66%–87% of observations with low bias (−0.58° to −5.24°) in more sheltered locations (Rst, Fxs, and Sca), where representation of directional waves is more critical. In exposed swell‐dominated locations (Blk, Prp), where directional variability is less, scatter reduces *R* ^*2*^ values (*R* ^*2*^ = 0.37–0.46), but bias and RMSE are comparable with sheltered locations.

**Table 1:** Validation Statistics Between Coastal Wave Buoy Observations and Modeled Wave Parameters

Site	*n*	*H*_s_			*T*_*z*_			*D*_p_		
		*R* ^2^	Bias	RMSE	*R* ^2^	Bias	RMSE	*R* ^2^	Bias	RMSE
“Blk”	184,316	0.94	−0.21	0.48	0.84	−0.42	0.76	0.46	−13.53	32.34
“Prp”	189,314	0.90	−0.06	0.31	0.67	−0.12	0.95	0.37	3.96	20.11
“Rst”	132,837	0.89	−0.03	0.21	0.55	−0.28	0.74	0.71	−1.87	25.01
“Fxs”	55,996	0.70	0.06	0.24	0.20	−0.35	0.72	0.87	−5.24	33.10
“Sca”	70,860	0.84	0.05	0.31	0.75	−0.32	0.70	0.66	−0.58	42.68

*Note*. Directional statistics computed using circular correlation and difference statistics (Berens, 2009).

Our analysis, including the use of peak spectral wave direction, relies on an assumption that bimodality is primarily asynchronous. A preliminary analysis indicated that synchronous bimodality, where the wave power of a secondary spectral peak is >5% that of the main peak, occurs <5% of the total time. The lowest synchronous spectral bimodality occurred in semi‐sheltered coastal regions typically associated with bi‐directional wave climates (S and E England). Therefore, the assumption of asynchronous bimodality is justified, and references to bimodality from herein refer to asynchronous bimodality. The extent of this asynchronous directional bimodality in the directional wave spectrum is shown by Wiggins, Scott, Masselink, Russell, and McCarroll (2019a) and Wiggins, Scott, Masselink, Russell, and Valiente (2019b) to relate to beach morphological response.

### 2.2 Atmospheric Data and Climate Indices

**Figure 3:**
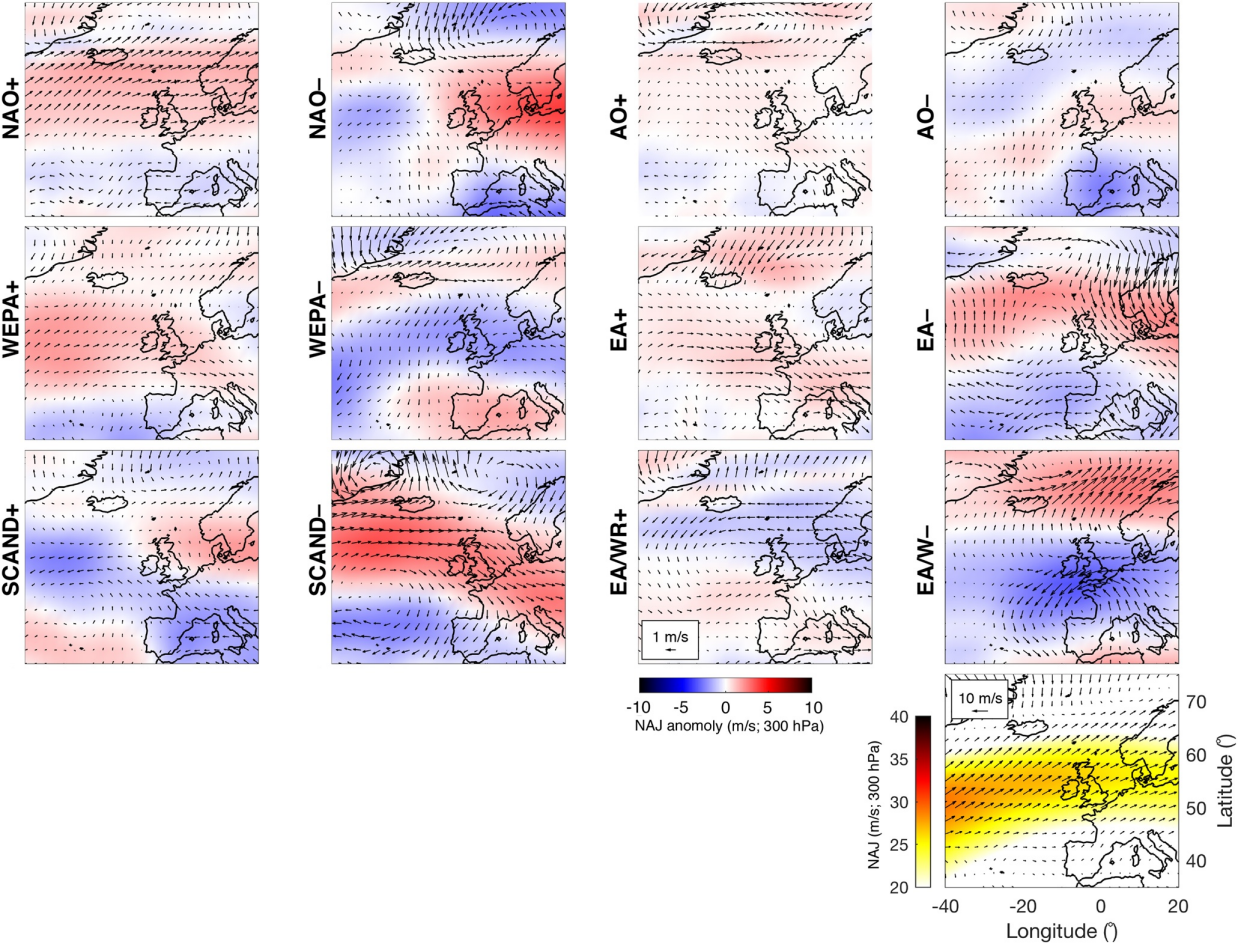
Atmospheric signature of all indices used in this study. Panels show sea surface wind (vectors) and 300 hPa winds relating to the North Atlantic Jet (NAJ; red/blue colors) anomalies from long term mean. Positive phase and negative phase of each index is addressed by averaging the 5 years with the largest and smallest index values over 1980–2017, respectively. Variability is displayed as anomalies from the long‐term mean (1980–2017). Long‐term mean sea surface wind field (vectors) and NAJ 300 hPa wind speed (color) are shown in bottom right panel for entire period.

Climate indices used in this study include the leading monthly teleconnection indices (NAO, EA, EA/WR, and SCAND) derived from rotated EOF analysis of the monthly mean standardized 500‐mb height anomalies in the Northern Hemisphere, as described in Barnston and Livezey (1987) and available for the period 1980–2017 (downloaded from the National Oceanic and Atmospheric Administration (NOAA) Climate Prediction Center; www.cpc.ncep.noaa.gov). In addition, we used the Western Europe Pressure Anomaly (WEPA), a climate index developed by Castelle, Dodet, Masselink, and Scott (2017) and computed as the normalized sea level pressure (SLP) gradient between Valentia (Ireland) and Santa Cruz de Tenerife (Canary Islands). Although the WEPA contains some variability of EOF‐based NAO, EA, EA/WR and SCAND, it was used as it provides a simple SLP‐based index that best explains winter wave height variability along the coast of western Europe, from UK to Portugal (52°N‐36°N), and which reflects a latitudinal shift of the Icelandic low/Azores high dipole. WEPA has also been shown in a number of studies to be most significantly correlated with winter wave heights and beach morphological changes in this region (e.g., Castelle, Dodet, Masselink, & Scott, 2018; Dodet, Castelle, et al., 2018; Wiggins, Scott, Masselink, Russell, & McCarroll, 2019a; Wiggins, Scott, Masselink, Russell, & Valiente, 2019b). We also used the Artic Oscillation (AO), a climate index representing the state of the atmospheric circulation of the Arctic computed by projecting the AO loading pattern to the daily 1,000‐mb height field anomaly above 20°N. The loading pattern of the AO is the leading mode from EOF analysis of monthly mean 1,000‐mb height for 1979–2000. While research has debated the usefulness of the AO, suggesting it is expressing the same physical phenomenon as the NAO (Itoh, 2008), it is included in this study due to the potential insights it may provide for subtle changes in directional wind‐wave fields in sheltered seas. These regional atmospheric signatures are highlighted in Figure 3, illustrating the association of +NAO and −SCAND with increased westerly winds at high latitudes, and −NAO and +SCAND linked to reduction in westerly airflow and blocking of the North Atlantic Jet (NAJ). It is also clear from Figure 3 that +WEPA represents a southward shift and strengthening of westerly sea surface winds in North Atlantic.

Atmospheric data were derived from the National Centers for Environmental Prediction (NCEP)/National Center for Atmospheric Research (NCAR) Reanalysis 1 project (downloaded from www.esrl.noaa.gov/psd/data/gridded/data.ncep.reanalysis.html), providing gridded (0.25° × 0.25°) 4 times daily (0Z, 6Z, 12Z, and 18Z) vector winds at 17 pressure levels from 1948 to the present (Kalnay et al., 1996).

## 3 Wave Climates in the UK and Ireland

**Figure 4:**
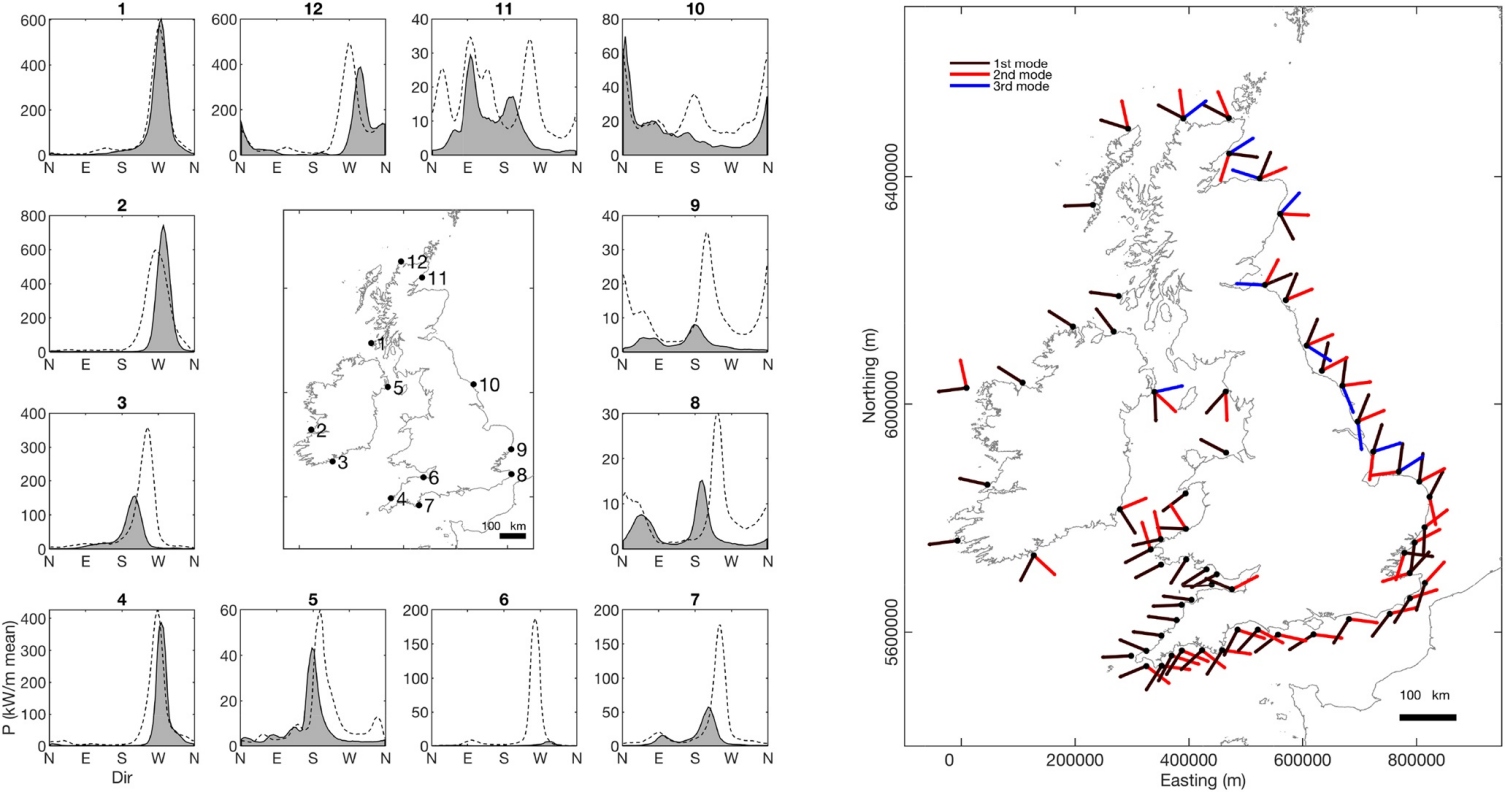
Left: Example winter wave climate data from each region of UK&I demonstrating the variability in directional multi‐modality. Insets show directional power distribution as 3‐hourly average kW/m in 5° bins for 1980–2017. Dashed lines are directional distribution at a location ∼40 km offshore of the coastal node, illustrating inshore modulation of the directional spectrum. Right: Directional modes taken from 1980–2017 cumulative winter (DJFM) wave power distribution for each wave node around the coast. Black is primary mode, red secondary and blue tertiary. Modes displayed are >5% of primary mode peak prominence. Mean primary peak half power width = 22°; therefore, approximately 68% (2*σ*) of peak distribution within 20° of peak.

Figure 1 provides an overview of the 63 inshore wave data nodes that span all the sheltered and exposed regions of the UK&I. These regions can be qualitatively separated into their geographic regions based on their wave exposure (annual/winter mean wave climate) and the characteristics of the directional modality of the wave climate (Table 1; Figure 4). Integrated winter wave climate is composed of months December through to March (DJMF), following previous analysis of climate indices (e.g., Castelle, Dodet, Masselink, & Scott, 2017). To assess directional multi‐modality throughout the UK&I and investigate the descriptive power that climate indices may have on the directional balance of alongshore wave power at these inshore locations, directional modes were extracted from the cumulative directional wave power distribution for each wave node around the coast and ordered by energy peak for 1980–2017 (Figure 4; right). Analysis of direction modality shows that 46 of the nodes (73%) have directionally multimodal wave climates where secondary modes (>5% of primary mode peak prominence) have >20° peak‐to‐peak separation. Across all nodes, mean prominent peak half power width = 22°; therefore, it can be estimated that >70% (∼2.35*σ*) of peak distribution is within 20° of peak.

The impact of exposure and coastal proximity on directional wave spectrum is demonstrated by the examination of the modulation from offshore (∼40 km distance) to the coast at a range of settings (Figure 3, left panel). At exposed, semi‐exposed and semi‐sheltered locations, mean variation in wave direction associated with the primary directional mode was 11°, 32°, and 40°, respectively; and 50% of coastal locations defined as directionally bimodal displayed a fundamentally different secondary mode (Figure 3, left panel).

In general, the most exposed west coast regions (winter‐averaged significant wave height *H*_s_ > 1.5 m and peak wave period *T*_p_ > 9 s; W Ireland and SW England & Wales) display the lowest levels of directional multimodality (20% and 33% of nodes, respectively) with NW Scotland being the exception (60%) due to a broad ocean swell window (Table 2; Figure 2). All nodes along the coasts of S & E England throughout the English Channel and North Sea coasts are directionally multimodal; these regions are also the most sheltered from open ocean swell and are the only regions where winter *H*_s_ < 1.2 m. Moving up the North Sea coast into NE Scotland, there is an increasing influence of northerly swell waves from the Arctic (winter *T*_p_ = 7.4 s), but wave climate bi‐directionality still dominates (100% of nodes) until the north‐facing coast of NW Scotland is reached. Coasts of W Wales, NW Wales & NW England, and E Ireland are located within the Irish Sea with varying influence of S‐SW Atlantic swell waves, resulting in much of the region being dominated by local wind wave regimes and local influence of coastal orientation (winter *H*_s_ = 0.9–1.3 m and *T*_p_ = 5.1–9.4 s; Table 1).

**Table 2:** UK Hindcast Wave Climate Statistics for the Period 1980–2016

Region	Nodes (*n*)	Annual *H*_s_ (m)	Annual *T*_p_ (s)	Winter *H*_s_ (m)	Winter *T*_p_ (s)	Bi‐directionality (%)
NW Scotland	5	2.0	10.3	2.7	11.8	60
W Ireland	6	2.0	10.3	2.6	11.9	20
SW England & Wales	12	1.3	9.4	1.7	10.8	33
W Wales	4	1.2	8.2	1.5	9.4	75
E Ireland	4	1.0	7.9	1.3	8.7	75
S England	13	0.9	6.7	1.1	7.5	100
NE Scotland	3	0.9	6.7	1.1	7.4	100
NW Wales & NW England	2	0.7	4.8	0.9	5.1	50
E England	14	0.7	6.1	0.9	6.6	100

*Note*. Nodes shown in Figure 1 are integrated into regions of similar characteristics and exposure. Winter wave statistics represent months December to March.

Initial wave climate analysis indicates that beyond the semi‐sheltered sites examined in Wiggins, Scott, Masselink, Russell, and Valiente (2019b) along the western English Channel coast, directionally bimodal wave climates exist in many inshore regions throughout the coasts of the UK&I. Of particular interest are nodes along the English Channel coast (S England) and southern North Sea coast (E England) where primary and secondary directional modes are from opposing directions with respect to the coastal shore‐normal, therefore having the greatest potential to influence coastal morphodynamics and shoreline plan‐shape rotation with respect to the directional balance of alongshore wave power (Figure 4).

## 4 Role of Atmospheric Indices

**Figure 5:**
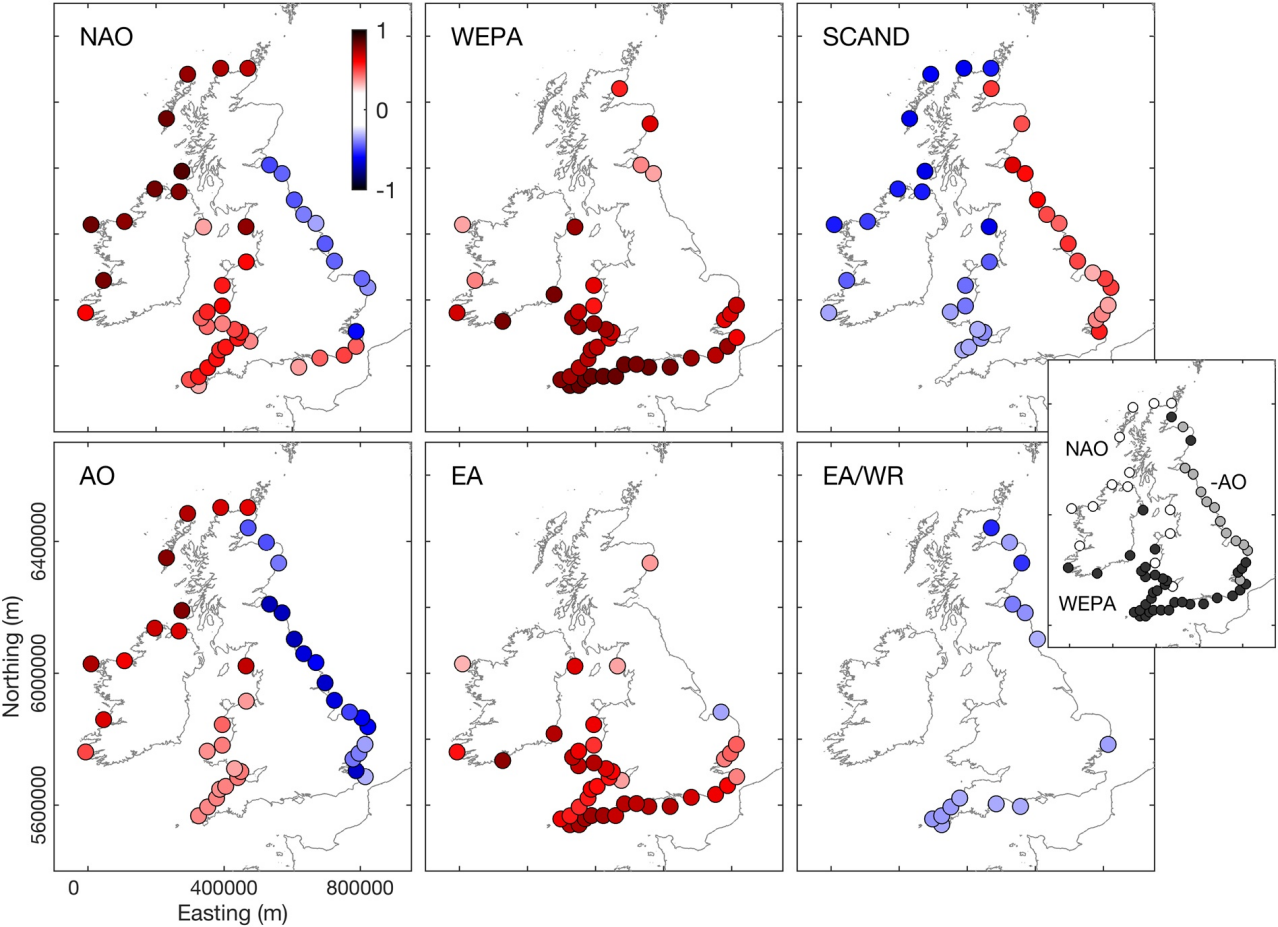
Correlations between mean winter wave power and six winter‐averaged atmospheric indices (NAO, WEPA, SCAND, AO, EA, and EA/WR) for 63 locations around the coast of the UK&I. Only locations where correlation coefficients (r) were significant at 95% level are shown (*p* < 0.05). Inset shows leading index for each node (white = NAO, black = WEPA, gray = AO).

To examine the relationships between long‐term atmospheric forcing and winter wave climate around the inshore waters of the UK&I, winter‐averaged NAO, WEPA, SCAND, AO, EA, and WR/EA indices were first correlated with total winter‐averaged wave power (1980–2017) for each node (Figure 5; hereafter all references to climate indices are DJFM winter‐averaged). Winter periods (DJFM) are used as they have the strongest relationship with climate indices and account for the largest and most significant beach morphological changes, both erosion and recovery (e.g., Dodet, Castelle, et al., 2018). Supporting the findings of Martínez‐Asensio et al. (2016) and Castelle, Dodet, Masselink, and Scott (2017), total winter‐averaged wave power was strongly and significantly (*p* < 0.05) positively correlated with the NAO along the Atlantic NW coasts (*r* = 0.6–0.83), but also significantly correlated with all exposed sites along the Atlantic SW coasts and within the Irish Sea, but with r‐values of 0.39–0.58 (Figure 5). This spatial relationship is matched by the AO, but is limited to the Atlantic W coasts with lower correlation coefficients (*r* = 0.5–0.8 in Atlantic NW coasts; *r* = 0.31–0.4 in Atlantic SW coasts). But, as observed by Castelle, Dodet, Masselink, and Scott (2017), the skill of the NAO in explaining total wave power reduces below southern Ireland (∼52°N), below which the skill of WEPA and EA dramatically increases. In fact, WEPA and EA are only significantly positively correlated with nodes in the southern half of the UK&I and, although WEPA and EA show a similar spatial correlation signature, WEPA significantly outperforms EA (15% increase in explanation of variance where there are significant correlations) showing the highest significant correlations along the Atlantic SW coast (*r* = 0.66–0.79) and W English Channel (*r* = 0.81–0.84). Negative (positive) SCAND also has significant skill in explaining annual mean wave power for the exposed Atlantic NW coast (and semi‐sheltered North Scotland coast) with r‐values between −0.32 and −0.63 (0.21 and 0.6). The SCAND spatial correlation and significance map is essentially the inverse of the AO. The stability of these relationships over time was explored by examining correlations before and after 2000. The spatial character of significant correlations remained the same for all indices, but strength varied. WEPA showed stronger correlations post 2000 and SCAND pre 2000. NAO correlations remained constant throughout both epochs.

In the context of previous ocean basin‐scale research, significant positive relationships with the NAO, AO, WEPA and EA, and also negative SCAND, for total wave power on Atlantic coasts were expected, but inshore regional scale (including shelter seas) analysis here reveals significant negative correlations with NAO/AO and positive correlations with SCAND along the mixed swell/wind‐wave dominated east‐facing North Sea coast (NAO *r* = −0.28 to −0.69; AO *r* = −0.41 to −0.71, SCAND *r* = 0.33–0.65) with AO showing the strongest relationship and level of significance (all sites significantly correlated at 95% level). This analysis shows that the EA/WR index provides the least descriptive power of all the indices tested.

**Figure 6:**
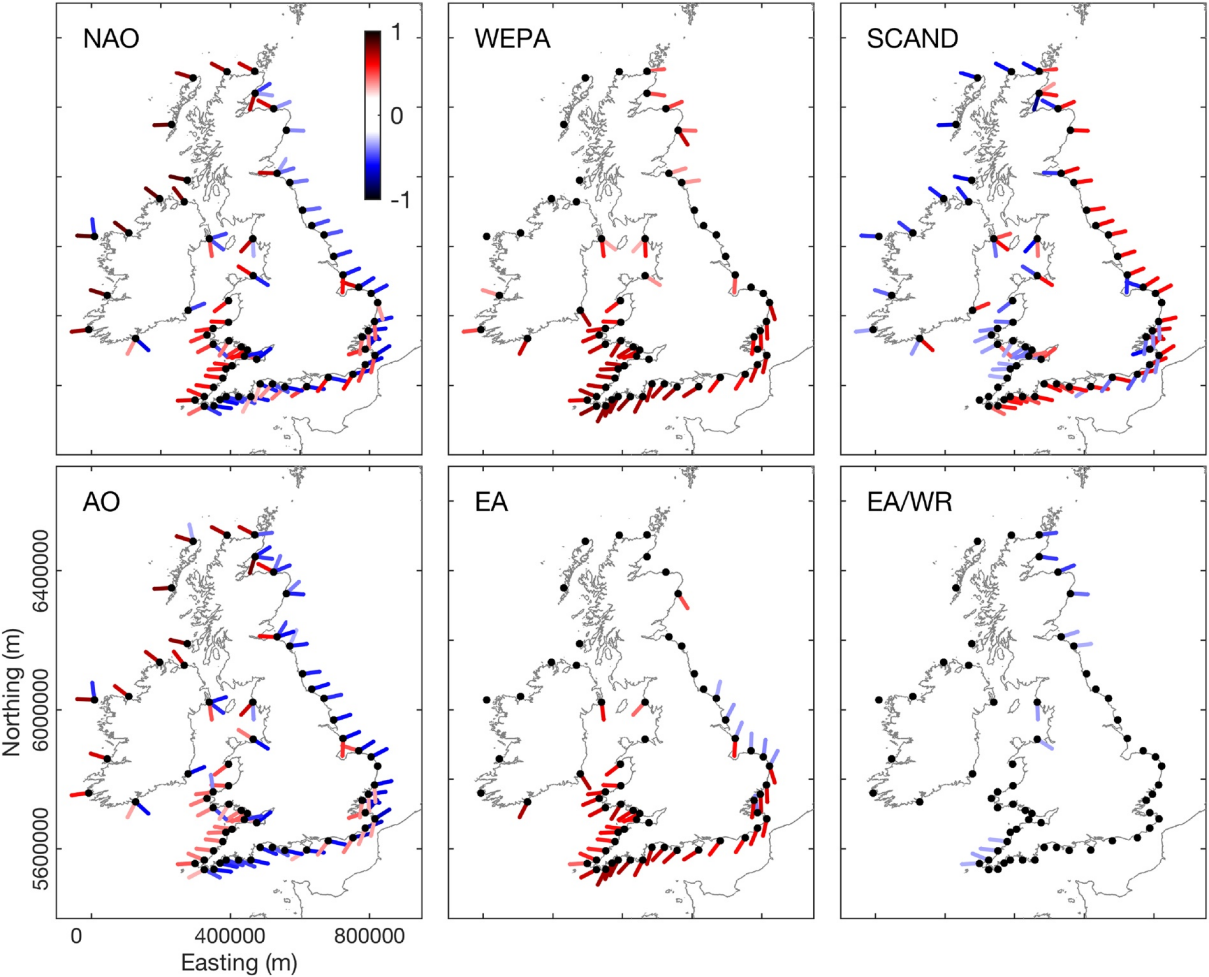
Relationship between winter‐averaged NAO, WEPA, SCAND, AO, EA, and EA/WR and directional winter‐averaged wave power (local wave directional window of ±20° for each node) for 63 wave nodes (black dots) around the coast of the UK&I (1980–2017). Colors are correlation coefficients (*r*), only results where *p* < 0.05 are shown.

To explore further the relationships with wave directional modes, winter (DJFM) cumulative wave power associated with local directional modes (angular window of 20° either side of power modal peak) were investigated as a function of all the indices (see Figure 6). Strikingly, results show that NAO (AO), WEPA (EA) and SCAND explain a significant amount of directional variability in winter‐averaged waves throughout UK&I, even in regions away from Atlantic swell waves where local wind wave regimes dominate. In many cases multiple directional modes have contrasting and highly significant atmospheric controls.

All nodes in the southern Ireland, English Channel and southern North Sea coasts (south‐facing) are strongly directionally bimodal (Figure 4), with coastal orientation suggesting dominance of alongshore sediment transport processes, which is supported by observations along this coast by Wiggins, Scott, Masselink, Russell, and Valiente (2019b). Analysis shows that WEPA (and EA to a lesser extent) significantly explains variability in winter‐averaged wave power for all southwesterly orientated (principal) wave directional modes (*r* = 0.58–0.77), accounting for the largest proportion of winter‐averaged wave power. In contrast, negative NAO explains variability in all easterly orientated wave modes (*r* = −0.6 to −0.76), with positive SCAND also contributing high correlations with easterly waves (*r* = −0.5 to −0.67). These findings mean the full winter‐averaged directional wave power balance is significantly explained by climate indices along this whole section of coast.

Beyond the south coast regions, easterly orientated wave modes throughout the bi‐directional North Sea region, characterized by short wind‐waves, showed significant relationships with negative NAO (and AO) where *r* = −0.35 to −0.57 (*r* = −0.5 to −0. 62) decreasing to the north. The strongest relationships in the North Sea region are found with the SCAND index, showing significant positive correlations with all easterly orientated nodes where *r* = 0.57–0.62. Interestingly, EA and AO, and NAO to a lesser extent, had some skill in explaining northerly swell waves entering this North Sea region, but only regionally. This northerly component is an important element of the northern North Sea coastal wave climate and is associated with a small swell window and is strongly impacted by coastal orientation. Whilst EA provided some skill in explaining northerly wave mode variability at six southern North Sea coast sites, significant *r*‐values only reached 0.39. AO and NAO showed some explanatory power for six nodes in the northern North Sea regions (E. Scotland) with significant *r*‐values between 0.33 and 0.59 (Figure 6).

Throughout the UK&I there are sheltered regions with complex coastal orientations (e.g., E Scotland, Irish Sea, and Bristol Channel) sheltered from significant swell‐wave contribution and that are dominated by a locally generated wind‐wave climate. The directional wave modes in these regions display a variety of significant relationships with indices dependent of spatial location and coastal orientation. Typically, easterly oriented short fetch nodes are related to negative NAO/AO, and positive SCAND; whereas westerly oriented short fetch nodes are related to positive NAO/AO, and negative SCAND.

In summary, winter‐averaged climate indices show strong and significant correlations with directional winter‐averaged wave power throughout the UK&I. Analysis indicates there are regionally coherent relationships between directional waves and various combinations of the leading climate indices. To examine the temporal variability and predictability of regional response characteristics, a quantitative connectivity‐based cluster analysis is first undertaken.

## 5 Characterization of Regional Response

**Figure 7:**
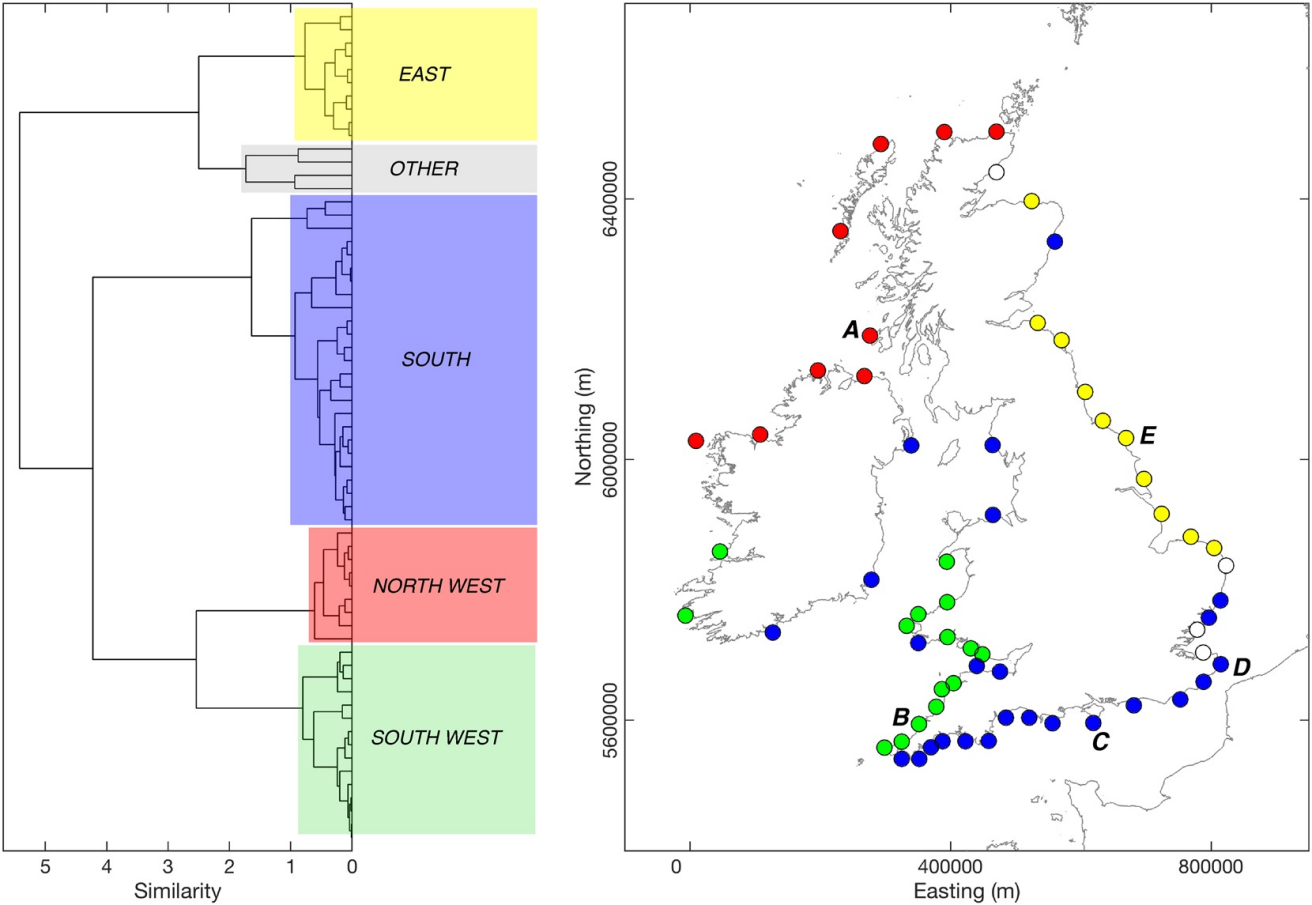
Regional classification of correlations between winter‐averaged directional wave climate response (mean wave power for dominant directional modes) and most significant winter‐average atmospheric indices NAO (EOF‐based), WEPA (station‐based), and SCAND (EOF‐based) for the period 1980–2017. Left: Dendrogram illustrating results of hierarchical agglomerative cluster analysis, using Ward's minimal increase of sum‐of‐squares Euclidian proximity method. Clear groupings are labeled. Right: Groupings from cluster analysis presented spatially, with nodes A–E representing case‐study sites from each region used for further analysis and visualization.

Regionally coherent relationships between the climate indices that had the strongest relationships with wave variability in UK&I were investigated through hierarchical cluster analysis. For all 63 nodes, the variables examined were mean directional winter wave power correlation coefficients with NAO, WEPA, AO, SCAND, and EA for the period 1980–2017. EA/WR was excluded at this stage as spatial correlations were weak and mostly statistically insignificant. EA and AO were retained as statistically they explained some of the variability in northerly waves in the North Sea. The clustering uses Euclidian‐based proximity to determine similarity between nodes where response variables are primary and potentially secondary directional mode correlations with winter‐averaged climate indices. The dendrogram shown in Figure 7 shows the results of the cluster analysis where Ward's minimum variance method is used to minimizes the total within‐cluster variance (weighted squared distance between cluster centers) at each step (Ward, 1963). The advantage of Ward's method is that it often provides a clear threshold number of groups where there is a large jump in group merging cost (e.g., above and below similarity level 1; Figure 7).

Four clear groupings were defined by this classification process (Figure 7). Two groups named South West and North West clearly represent the largely directionally uni‐modal west coasts that are dominated by ocean swell waves. A group named South represents nodes that are bi‐directional in nature and largely located on the southern and western regions of the UK&I. The final major group named East also represents bi‐directional wave climates, but the region is limited to the eastern North Sea coast and only consists of sites exposed to northerly swell waves. To elucidate the relationships between climate indices and directional wave power for each group, example nodes (A–E; Figure 7) representing case study sites from each cluster, were examined further.

**Figure 8:**
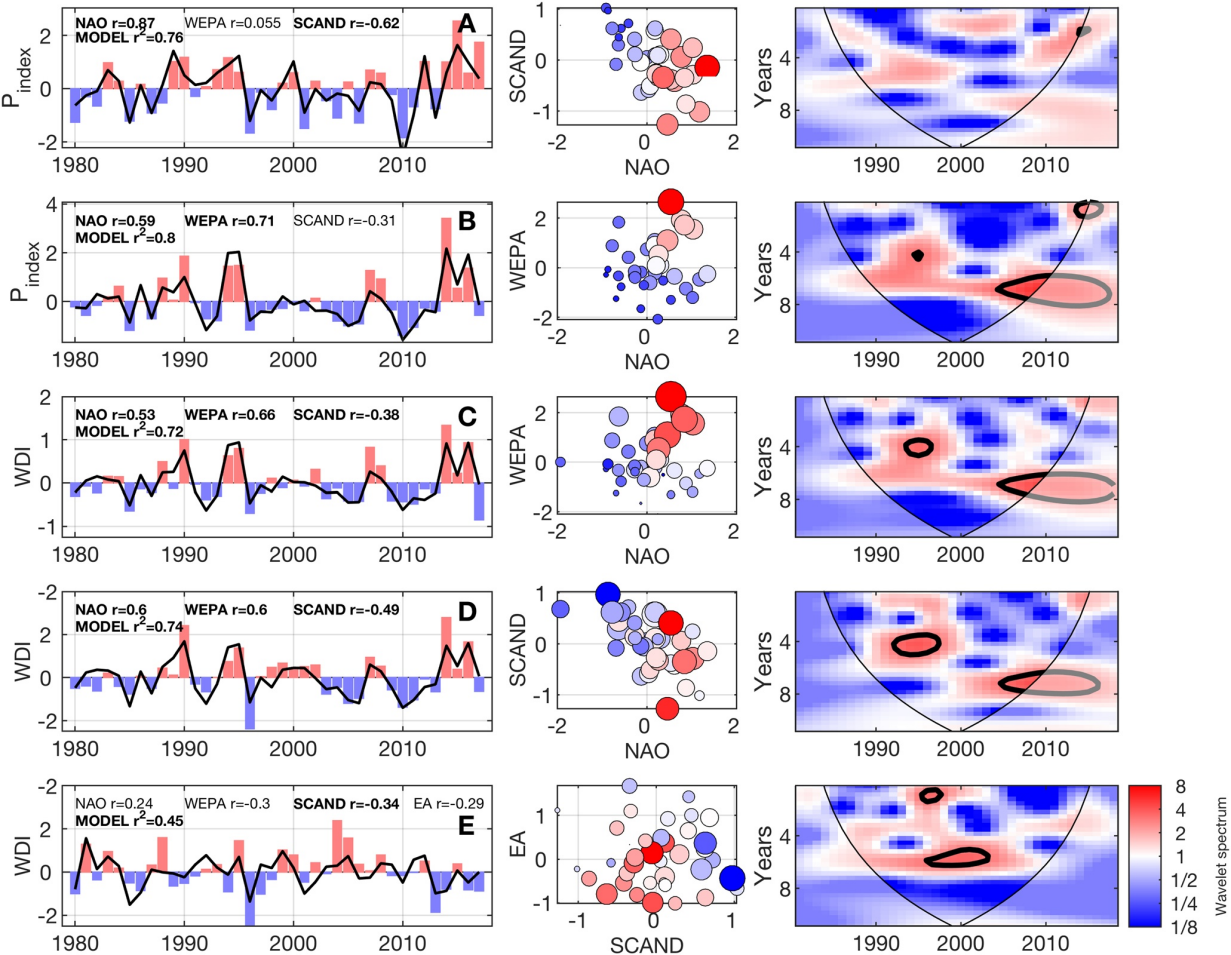
Temporal variability of standardized winter‐averaged wave power (P_index_; uni‐directional regions) and wave direction (WDI; bi‐directional regions) for case study sites from each of the 4 major classified regions are shown. Locations of Sites A–E are shown on Figure 7. Left panels show 1980–2017 wave power (P_index_; top two panels A and B) and winter‐averaged WDI timeseries (bottom three panels C–E) for each winter season as red/blue bars, for each site correlations (*r*) with winter‐averaged NAO, WEPA, and SCAND are shown (bold is significant at the 95% level). In addition, stepwise multiple linear regression (SMLR) model *R* ^*2*^ values are shown (bold is significant at the 95% level) and model prediction line is shown (bold black). Middle panels show winter‐averaged P_index_ (top two) and WDI values (bottom three) within a 2D parameter space of the statistically strongest descriptive variables from SMLR for each site. Color is P_index_ or WDI (low/high = blue/red, white = zero), bubble size is standardized winter wave power. Right panel shows local wavelet spectrum normalized by the variance of associated power and wave direction index. In wavelet panels, the 5% significance level against red noise is contoured in bold black line and the cone of influence is delimited by the fine black line.

Figure 8 examines the atmospheric indices controlling the wave climate at each case study site. Where the wave climate is directionally uni‐modal, the standardized long‐term (1980–2017) winter‐mean wave power timeseries is examined through a wave power index (P_index_). Where the wave climate is significantly directionally bimodal, a wave power directionality index (WDI) is computed following Wiggins, Scott, Masselink, Russell, and McCarroll (2019a), which represents the winter‐averaged standardized wave power balance between two opposing wave directional modes, shown by Wiggins, Scott, Masselink, Russell, and Valiente (2019b) to correlate with observed beach rotation. At each node, an index of the relative balance between winter wave power contributions from the two modal directions is computed, using the equation:

(1) $$\text{WDI}=\frac{\left(P_{1}-P_{2}\right)-\bar{\left(P_{1}-P_{2}\right)}}{\sigma \left(P_{1}-P_{2}\right)}$$

where $\left(P_{1}-P_{2}\right)$ is the residual wave power between the first (prominent) and second directional wave power modes, $\bar{\left(P_{1}\;-\;P_{2}\right)}$ is the long‐term mean and $\sigma \left(P_{1}-P_{2}\right)$ is the long‐term standard deviation of that difference. High positive values of WDI indicate that the primary directional mode is more prevalent than the long‐term average, whereas high negative values indicate that the wave climate has a higher proportion of the secondary directional mode than average.

For all bi‐directional example sites shown in Figure 8, WDI time‐series show significant temporal variability over the 37‐year period. WDI within the South region (Sites C and D; Figure 8 left panel) displays a consistent 5–8 year cyclicity that can be observed from the continuous wavelet transform analysis shown in Figure 8 (right panels) computed as in Castelle, Dodet, Masselink, and Scott (2018) using a scaled and normalized Morlet function, following Grinsted et al. (2004). Periodicity is strongest and most significant since 2005. This corresponds to the periodicity observed in the WEPA index by Castelle, Dodet, Masselink, and Scott (2018) through similar wavelet analysis. WDI at these South group sites is significantly positively correlated with WEPA, NAO, and SCAND, with WEPA having a stronger correlation in the west of the region (Site C) and NAO/SCAND increasing in importance the east (Site D). The case study site from the East group in the mid North Sea coast (Site E) shows a significant 5–6 year periodicity in WDI, and shows weak or insignificant correlations to the climatic indices tested, with the exception of SCAND. Example sites from North West and South West groups, which are directionally uni‐modal, P_index_ are strongly correlated with NAO and WEPA, respectively, and are therefore dominated by any periodicity of these indices. Unlike WEPA, there is little coherent periodicity displayed within NAO dominated sites (Site A) and there are high degrees of variability over the period 1980–2017, with the only significant timescales of ∼2 years occurring post 2010 (Figures 8a and 8b; right panel). This lack of NAO periodicity has previously been demonstrated by Barbosa et al. (2006) and Castelle, Dodet, Masselink, and Scott (2018).

It is clear that the inter‐annual variability of winter‐averaged directional wave power throughout the UK&I is well explained by a range of individual indices, but it is useful to explore whether or not when combined they have greater predictive skill, particularly for bi‐directional regions. Empirical stepwise multiple linear regression models were computed for each example site using combinations of the leading indices for each location based on correlation analysis and their predictive skill (Table 3). A full model with all terms included (including interactions) is refined in each case through adding/removing terms that are lower/higher than a *p*‐value tolerance, until model reaches stability (Figure 8; left panels).

**Table 3:** Stepwise Multiple Linear Regression Models for Winter‐Mean Wave Power (Sites A and B) and WDI (Sites C, D, and E) as a Function of Combined Climate Indices

Site	Index	Weight	RMSE	*R* ^*2*^	*p‐*value
A (North West)	NAO	1.29	0.50	0.76	2 × 10^−12^
B (South West)	NAO*WEPA	0.32	0.46	0.8	4 × 10^−12^
C (South)	NAO*WEPA	0.33	0.56	0.72	9 × 10^−9^
	SCAND	−0.49			
D (South)	NAO	0.47	0.54	0.73	2 × 10^−9^
	WEPA	0.59			
	SCAND	−0.68			
E (East)	NAO*SCAND	0.74	0.82	0.45	25 × 10^−3^
	WEPA*SCAND	−1.18			
	SCAND*EA	1.47			

*Note*. Coefficients are standardized weightings of significant variables used in the model.

For all sites, except Site A in the North West group, the combination of multiple indices in a linear model outperformed the use of a single index. The model elements are presented in Table 2. Both sites from exposed uni‐directional groups (North West and South West) demonstrated very skillful reconstructions of winter wave power with combinations of NAO and WEPA with *R* ^*2*^ values exceeding 0.75. Specifically, Site B from the South West group demonstrated the improved skill gained by utilizing the product of two indices (*R* ^*2*^ = 0.8). WDI from the South group (Sites C and D) was skillfully predicted utilizing a combination of NAO, WEPA and SCAND. The discriminatory power of these combined indices is visualized in NAO/WEPA and NAO/SCAND parameter space (Figure 8; middle panels) demonstrating, for example, that at Site C all winters experiencing positive WDI occurred when both NAO and WEPA were positive. Likewise, SCAND when combined with EA at Site D accounts for a greater proportion of the variance in WDI. Finally, in the East region (Site E), where SCAND was the only index significantly correlated with WDI (*r* = 0.35), the combination of NAO, WEPA and SCAND with EA generated a linear model with significant explanatory skill (*R* ^*2*^ = 0.45).

**Figure 9:**
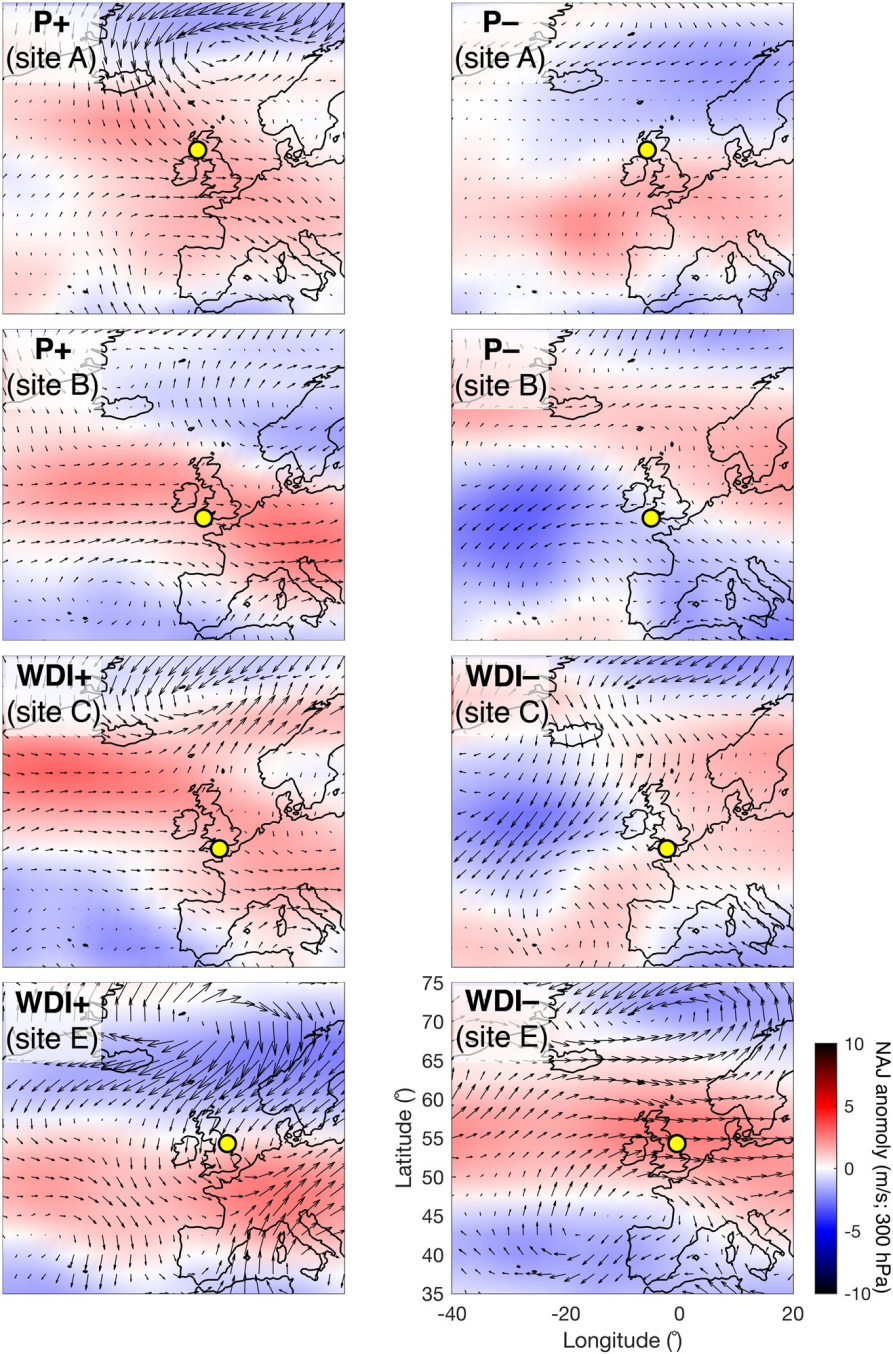
Atmospheric expressions of the directional wave power indices (P_index_ and WDI) for example sites (yellow circles; A, B, C, and E) in each response group (North West, South West, South, and East; top to bottom). Methodology and symbology as per Figure 3.

The atmospheric expression of modeled P_index_ and WDI values from case study sites for each classified region (1980–2017) are explored in Figure 9 by taking the highest and lowest 5 years for each P_index_ (the same methodology as Figure 3). The highest five P_index_ years (P+) at North West and South West example sites (Sites A and B) reflect the atmospheric signature NAO+ and WEPA+, respectively (Figure 3). In both sites (in particular Site B), the influence of the positive and negative SCAND patterns can be seen in both surface winds and the NAJ anomaly. The P+ anomaly seen in the North West group case (Site A) is expressed as an increase in northwesterly surface winds above 55°N and a northward shift of the NAJ, while in the South West group case (Site B) P+ corresponds to a clockwise rotation of the NAJ with a striking southward dip below 50°N over UK&I and NW Europe; this NAJ southerly shift is somewhat characteristic of WEPA+ and SCAND−.

Certainly, the most significant difference in P+ between the North West and South West group cases is the increase in westerly surface winds below 53°N (southern Ireland) for the latter when compared to a NAO+ (P+ Site A) scenario. This observed relationship between the NAJ and NAO is unsurprising and as year‐to‐year variability in the NAO is known to describe the state of the Atlantic jet stream which is directly related to near‐surface winds across North America, Europe, and other regions around the Atlantic Basin (Scaife, Arribas, et al., 2014).

For the bi‐directional South group (Site C), the WDI+ expression is strongly reflected in WEPA+ with increased westerly surface winds over the southern half of the UK&I. The WDI− expression is one of increased southeasterly anomalies in surface winds throughout the English Channel and North Sea and a dramatic reduction in westerly winds in the North Atlantic, limiting southwesterly swell in this region; this is associated with an Atlantic shift of the NAJ to the north (>65°N) or south (<45°N) away from the western approaches and reflects patterns of SCAND+ and NAO−.

The WDI+ atmospheric expression from the East group (Site E) shows a very strong latitudinal shift of the NAJ below 50°N represented by a reduction in NAJ windspeeds of ∼5 ms^−1^ throughout the north and increase to the south. A strong northeast surface wind anomaly (>2 ms^−1^) above 50°N throughout the North Sea and NE Atlantic represents a condition that may be representative of increased northerly swell wave propagation into the North Sea. This pattern is not clearly reflected in NAO, WEPA or SCAND, but can be partially reflected in the EA‐pattern (Figure 3). The signature of WDI− in the East group is even less clear reflecting the complexity of the wave climate and highlight issues/limitations of trying to use climate indices to predict directional wave climate in this region (East).

## 6 Discussion and Conclusions

This study has shown there exists strong and significant connections between leading climate indices in the North Atlantic and winter‐averaged wave power in inshore regions, both in exposed and (semi)‐ sheltered coastlines. For the first time, we have demonstrated the full extent of directionally bimodal inshore wave climates around the coast of the UK&I, as well as the significant role climate indices play in describing the physical processes that drive their inter‐annual variability over four decades.

It is well established that climate indices like ENSO in the Pacific and AO/NAO in the Atlantic are leading modes of atmospheric variability and strongly affect winter wave energy (Bromirski et al., 2013; Castelle, Dodet, Masselink, & Scott, 2017; Dodet, Bertin, & Taborda, 2010), and recent studies have also shown how extreme phases can lead to large‐scale coastal erosion and shoreline change (Barnard et al., 2015; Dodet, Castelle, et al., 2018; Masselink et al., 2016). But, there is now a growing base of evidence highlighting the role leading modes of climate variability also have in controlling wave direction and associated longshore sediment re‐distribution and shoreline rotation at the coast (e.g., Goodwin et al., 2016; Silva et al., 2012; Splinter et al., 2012; Wiggins, Scott, Masselink, Russell, & Valiente, 2019b). In extreme cases, as shown by Wiggins, Scott, Masselink, Russell, and McCarroll (2019a) when studying the impacts of the extreme storm wave events of the 2013/14 winter in Northwest Europe, resultant embayment rotation in semi‐sheltered regions can lead to extreme coastal vulnerability and infrastructural failure.

The analysis of 37 years of hindcast modeled wave data from 63 inshore nodes around the entire coast of UK&I has shown that 73% of studied sites have directionally bimodal wave climates (where secondary modes are >5% of primary mode peak prominence), with all sites within the English Channel and North Sea regions found to be directionally bimodal. Of specific relevance to coastal dynamics, primary and secondary modes within the English Channel and the southern North Sea coasts are opposing with respect to the coastal normal and have the greatest potential to influence coastal morphodynamics due to the importance of the directional balance of alongshore wave power. This is evidenced by Wiggins, Scott, Masselink, Russell, and Valiente (2019b) through the examination of a decade of beach observations and embayment rotation along the south coast of England. Importantly, the analysis of these wave directional modes as a function of leading climatic indices found that combinations of the NAO, WEPA, SCAND, and EA significantly explained the directional variability in modeled winter‐averaged directional wave power throughout all coasts and modal directions within the UK&I. It is important to acknowledge here that these relationships are based on modeled wave data. While the model hindcast data were shown to statistically correlate well with observations at the coast (Section 2.1), the findings of this study are only relevant to the part of the variance of observations that the model can describe.

The seasonal variability of directional winter wave power throughout the UK&I also demonstrated clear regional coherence. Cluster analysis of all coastal nodes driven by winter‐averaged directional wave correlations with NAO, WEPA, SCAND, AO, and EA for the period 1980–2017 identified four key regions that had distinct responses to atmospheric variability. The classes were strongly defined by wave exposure, coastal orientation and latitude; and the regional classes were closely related to the impact that sea surface and NAJ wind expressions of related indices could have on directional waves. Empirical multiple linear regression for regional examples from each class demonstrated significant skill (*R* ^*2*^ = 0.5–0.8) for both uni‐ and bi‐directional sites. Specifically, skill in predicting WDI at bi‐directional sites was significantly improved where different indices were correlated with contrasting wave directional modes and variability in the winter‐averaged balance of directional wave power (WDI index) was not fully explained by an individual index, rather a combination of multiple indices (Figure 8; middle panel). Similar observations were made by Woollings et al. (2010, 2012) who had some success in explaining the location and strength of the NAJ with a statistical mixture model defined by the NAO and the EA (similar to WEPA). It is expected that a non‐linear statistical modeling approach using multiple indices or dominant atmospheric modes from PCA may in future provide stronger descriptive relationships and improve predictive skill.

If strong relationships can be established between large scale seasonal atmospheric behavior and wave directionality along both exposed and more sheltered coasts that are dominated by longshore sediment transport processes, then these relationships can facilitate the extension of our understanding of past (historic) coastal behavior. This is because atmospheric sea level pressure (and proxy) records (e.g., Camus et al., 2014; Luterbacher et al., 1999) and modeled wave reanalysis' (e.g., Santo et al., 2015), whist containing inherent uncertainties, are much longer than those of sea surface waves or coastal morphological observations (<40 years; e.g., Turner et al., 2016). The extent of variability and periodicity of each of the leading atmospheric indices also provides insights into coastal geomorphological response of beaches past and present (e.g., Castelle, Dodet, Masselink, & Scott, 2018). This was shown recently by Dodet, Castelle, et al. (2018), where coastal (beach/dune) response to the disturbance of extreme winters can be multi‐annual and the rate and extent of beach recovery is critically related to subsequent winter wave power and directionality.

A key question is whether these relationships between climate indices and beach morphodynamics can lead to improved forecasts of coastal change. Recently, Hilton et al. (2020) demonstrated that a season‐ahead shoreline prediction model (ShorFor) could be significantly improved when a synthetic wave generation algorithm was informed by a priori knowledge of relevant indices (NAO or WEPA for SW England). For these kinds of approaches to have value, there need to be skillful long‐range forecasts of climate indices and atmospheric circulation. Until recently, long‐range forecast systems showed only modest skill in “season ahead” predictions of Atlantic winter climate and the NAO, partially due to the lack of response in the extratropical atmospheric circulation to long‐term predictive variability of the ocean (Smith, Scaife, & Kirtman, 2012). However, Scaife, Arribas, et al. (2014) recently demonstrated significant skill (*r* = 0.6) in predicting the NAO when initialized a month before the onset of winter and argued that greater ensemble sizes would lead to greater skill (Scaife, Arribas, et al., 2014). The NAO is described well in climate models, both in terms of interannual variability and mean state; and seasonal forecasts contain ensemble variability that matches the observations despite errors in the amplitude of forecast signals (Baker et al., 2018; Scaife, Arribas, et al., 2014). In terms of forecasts, large steps have been taken in recent years toward improved skill in forecasts, and Scaife, Arribas, et al. (2014) recently demonstrated significant skill (*r* = 0.6) in predicting the NAO when initialized a month before the onset of winter. These forecasts are now being used in other parallel applications for transport (Palin et al., 2016), hydrology (Svensson et al., 2015) and energy (Clark et al., 2017).

**Figure 10:**
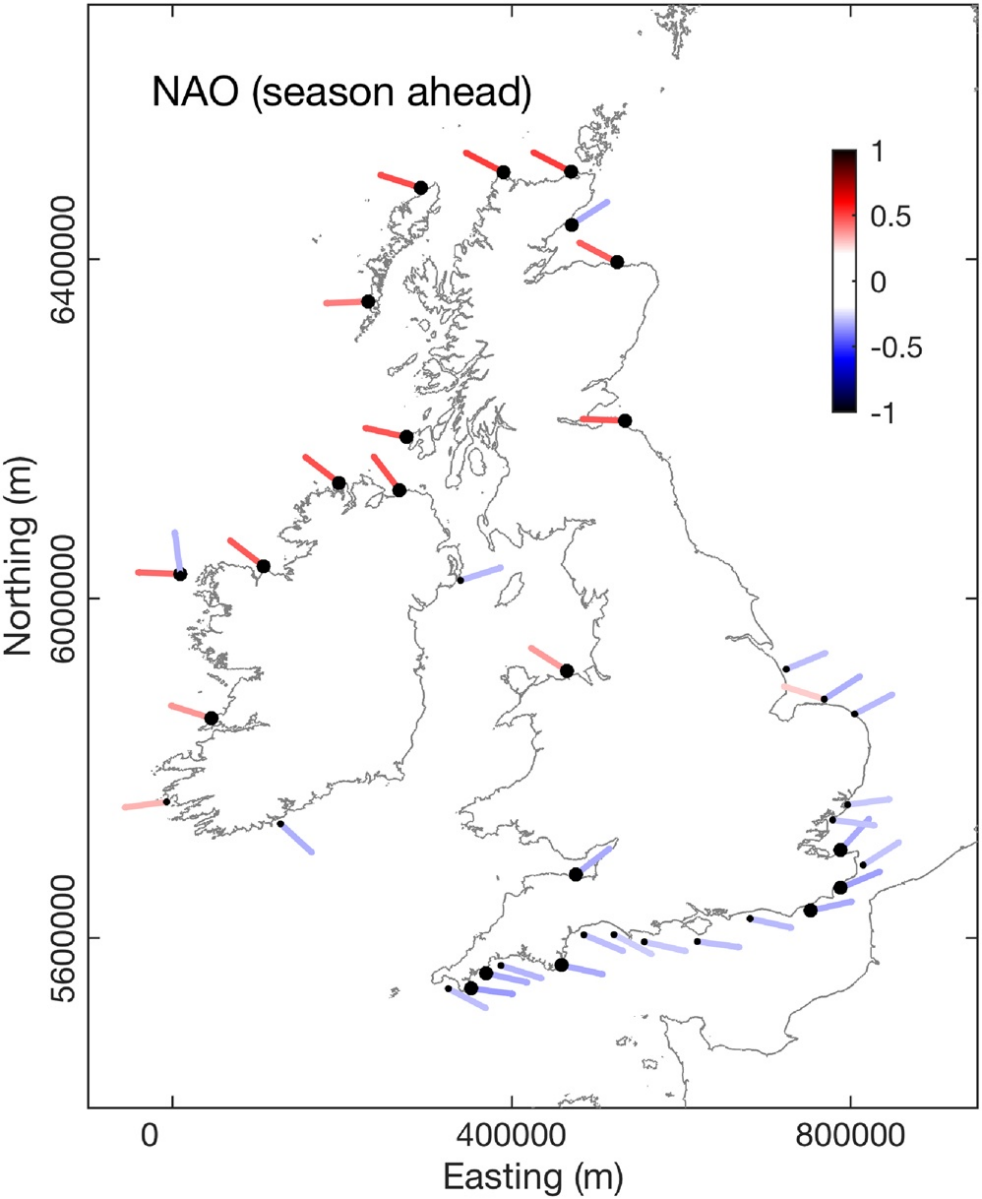
NAO “season ahead” forecast: (upper) relationship between season‐ahead forecast winter NAO and directional winter‐averaged wave power (local wave directional window of ±20° for each node) for 63 wave nodes around the coast of the UK&I (1980–2016). Colors are correlation coefficients (*r*), only results where *p* < 0.1 are shown, larger black dots represent *p* < 0.05.

To explore the state‐of‐the‐art we examined the season‐ahead retrospective forecasts (hindcasts) of the winter‐averaged NAO and WEPA, provided by version 3 of the Decadal Prediction System (DePreSys3) of the UK Met Office, as outlined in Dunstone et al. (2016). Forecasts were run for winters 1980–2016, with hindcasts initialized on November 1 each year. There is a significant correlation skill score for the season‐ahead forecasts of the observed pressure‐dipole based NAO *r* = 0.57 (*p* < 0.05), but there is poor correlation skill score for the season‐ahead forecasts of observed pressure‐dipole based WEPA (*r* = 0.13, *p* >> 0.05). If the NAO forecast is then examined in the context of the modeled wave data used in this study, uni‐directional nodes between southwest Ireland and north Scotland are significantly correlated (at 95% level) with forecast NAO (1980–2016) where *r* = 0.37–0.52 (Figure 10). This relationship disappears for the South West group, where variability is more strongly linked to WEPA. Correlation scores for the bi‐directional groups South and East showed significant inverse correlations (at 95% level) with secondary mode easterly waves at six sites along the English Channel coast, specifically those sites with a southeasterly orientation. At the 90% level, the easterly wave component of wave climate throughout English Channel and southern North Sea is significantly negatively correlated with forecast NAO. For sites in the South and East groups, this skill does not translate into predictability of WDI due to the strong influence of WEPA explaining the primary southwesterly directional waves. To the authors' knowledge, these findings are the first demonstration of skillful “season ahead” forecasts for modeled inshore directional winter wave climate.

**Figure 11:**
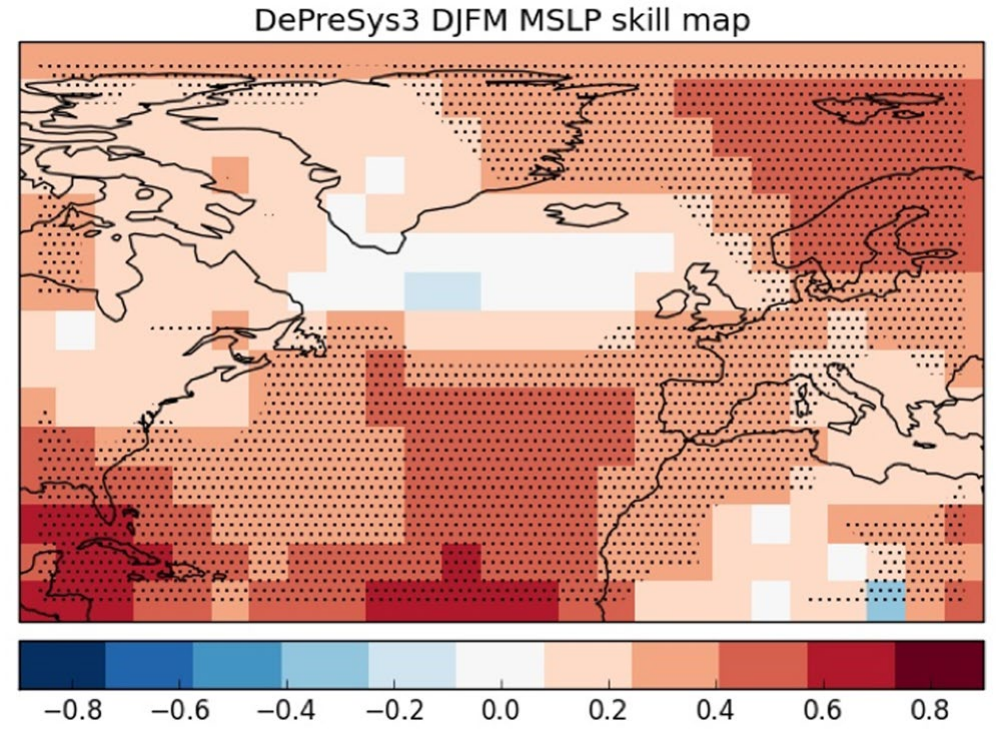
Spatial distribution of skill (correlation) for UK Met Office Decadal Climate Predication System 3 (DePreSys3), predicting the season ahead winter (DJFM) mean sea level pressure. Stippled regions are significant at the 5% level according to a Student's *t*‐test.

As noted by Scaife, Arribas, et al. (2014), much of the forecast skill of the atmospheric model is derived from the ability to predict the NAO and this is highlighted by the lack of model skill in regions where NAO influence is weak. It is therefore unsurprising that indices explaining secondary modes of variability like WEPA are currently not well predicted (*r* = 0.13; *p* = 0.353). An examination of the skill map for predicting DJFM Mean Sea Level Pressure (MSLP, Figure 11) confirms that, on average, DePreSys3 shows little MSLP skill over UK&I (located in the region of high uncertainty due to the NAJ variability). Instead, significant skill is found to the South (over the Azores/Southern Europe) and to the North (north of Iceland and over Scandinavia). While this is a limitation for the UK&I and indices like WEPA, this does suggest that the implementation of the approach used in this study in regions of greater atmospheric model skill may yield stronger results. Encouragingly, recent studies have highlighted the potential for future improvements in seasonal (Athanasiadis et al., 2016) and decadal (Smith, Eade, et al., 2019; Smith, Scaife, Eade, Athanasiadis, et al., 2020) forecast skill through increased ensemble sizes, citing the “signal to noise paradox,” which identifies that climate models (particularly for the Atlantic) are better able to predict their observed counterparts than their weak signal‐to‐noise ratios may suggest, meaning there may be much more potential predictability of indices like the NAO with larger ensemble sizes (Scaife & Smith, 2018).

In summary, this study has demonstrated that over 70% of inshore wave climates analyzed throughout the United Kingdom and Ireland were directionally bimodal, and shown that combinations of winter atmospheric circulation indices NAO, WEPA, SCAND and EA are significantly correlated with directional wave climates in all regions. Regression models using multiple winter atmospheric indices enabled skillful reconstructions of directional wave climate in all regions (*R* ^*2*^ = 0.45–0.8) and we demonstrated for the first time the significant explanatory power of leading winter‐averaged atmospheric indices for directional wave climates, and show that leading seasonal forecasts of the NAO skillfully predict wave climate in some regions. Future work should focus on improving statistical relationships between atmospheric indices and wave directional power spectrum globally; and optimizing long‐term forecasts of coastal directional wave climate using state‐of‐the‐art atmospheric forecasts, especially in regions where forecast skill is high.

## Data Availability

The United Kingdom and Ireland directional wave climate data were from the UK Met Office 8‐km WAVEWATCH III third‐generation spectral wave model (version 3.14; Tolman, 2009). This data set is not publicly available due to commercial restrictions, but can be sourced from Dr. Andy Saulter (andrew.saulter@metoffice.gov.uk) for specific research purposes. EOF‐based climate indices used in this study are publicly available for the period 1980–2017 (National Oceanic and Atmospheric Administration (NOAA) Climate Prediction Center; www.cpc.ncep.noaa.gov). The Western Europe Pressure Anomaly (WEPA) climate index (1943–2018) developed by Castelle, Dodet, Masselink, and Scott (2017) is publicly available online via the University of Plymouth PEARL open access research repository (http://hdl.handle.net/10026.1/15509). Season‐ahead retrospective forecasts (hindcasts) of the winter‐averaged December to March (DJFM) NAO and WEPA are not publicly available due to commercial restrictions, but can be sourced from Prof. Adam Scaife (adam.scaife@metoffice.gov.uk) for specific research purposes.
